# Functional bracing for delayed union of a femur fracture associated with Paget's disease of the bone in an Asian patient: a case report

**DOI:** 10.1186/1749-799X-5-33

**Published:** 2010-05-12

**Authors:** Iori Takigami, Akira Ohara, Kazu Matsumoto, Masashi Fukuta, Katsuji Shimizu

**Affiliations:** 1 Department of Orthopaedic Surgery, Gifu University Graduate School of Medicine, Gifu, Japan; 2 Department of Orthopaedic Surgery, Matsunami General Hospital, Gifu, Japan

## Abstract

Paget's disease of the bone is a common metabolic bone disease in most European countries, Australia, New Zealand, and North America. Conversely, this disease is rare in Scandinavia, Asia, and Africa. In Japan, it is extremely rare, with a prevalence of 0.15/100000. Paget's disease is a localized disorder of bone remodeling. Excessive bone resorption and abnormal bone formation result in biomechanically weakened bone and predispose patients to fracture. Delayed union and non-union of fractures have been reported in patients with Paget's disease. Therefore, open reduction and internal fixation of fractures has been recommended to prevent such complications. Here we report an unusual case of a 63-year-old Asian woman with delayed union of a femur fracture secondary to Paget's disease, which was treated successfully by functional bracing.

## Introduction

Paget's disease of the bone was first described by Sir James Paget in 1877. It is a well documented metabolic bone disorder in European countries and the United States, with a reported incidence of 3-4% in the adult population [1-3]. Interestingly, it is extremely rare in Africa and Asia, and rarely occurs in Japanese individuals [3-6].

Although the etiology of Paget's disease remains unclear, it is characterized by increased bone resorption, bone formation, and remodeling. The axial skeleton is frequently involved and the bones most commonly affected include the pelvis (70%), femur (55%), lumbar spine (53%), skull (42%) and tibia (32%) [7]. Increased bone turnover and remodeling leads to altered bone quality, thickening, enlargement, and deformity. Paget's disease is associated with significant disability, impaired quality of life and a variety of complications, such as osteoarthritis, pathological fracture, and nerve compression syndromes. Here we present an unusual case of delayed union of a femur fracture secondary to Paget's disease in an Asian patient, which was treated successfully by functional bracing.

## Case presentation

**Figure 1:**
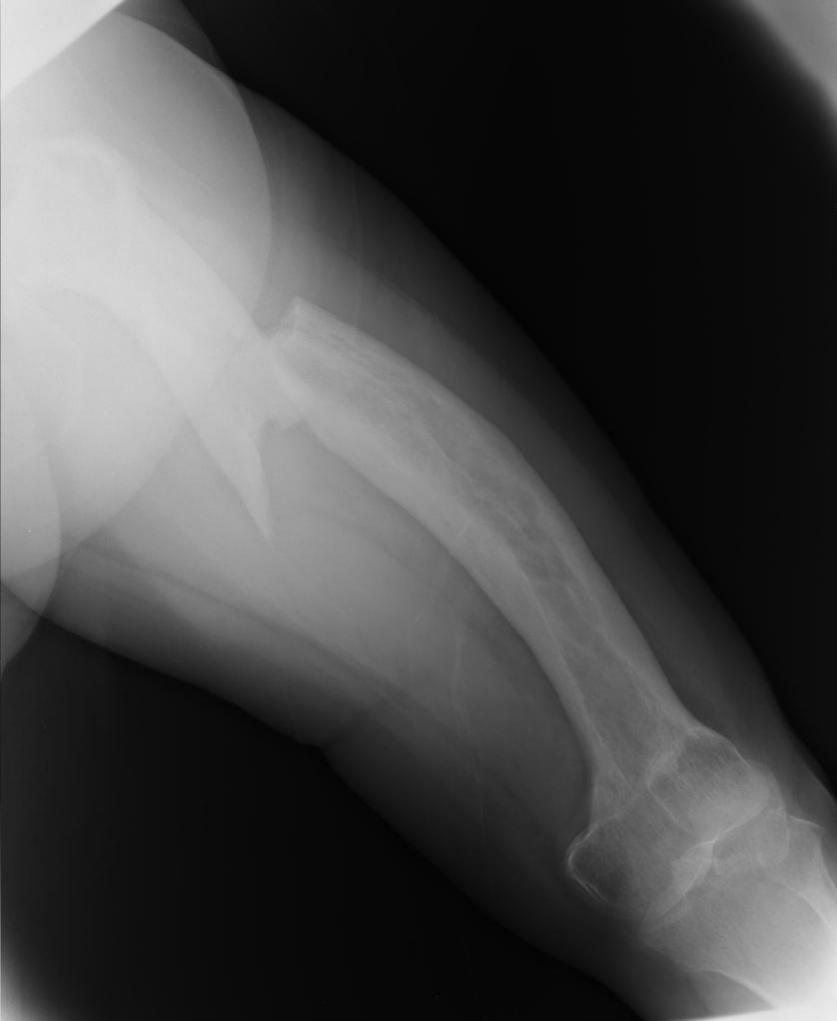
**Transverse fracture at the junction of proximal and middle thirds, and Paget's disease involving the entire femur**.

**Figure 2:**
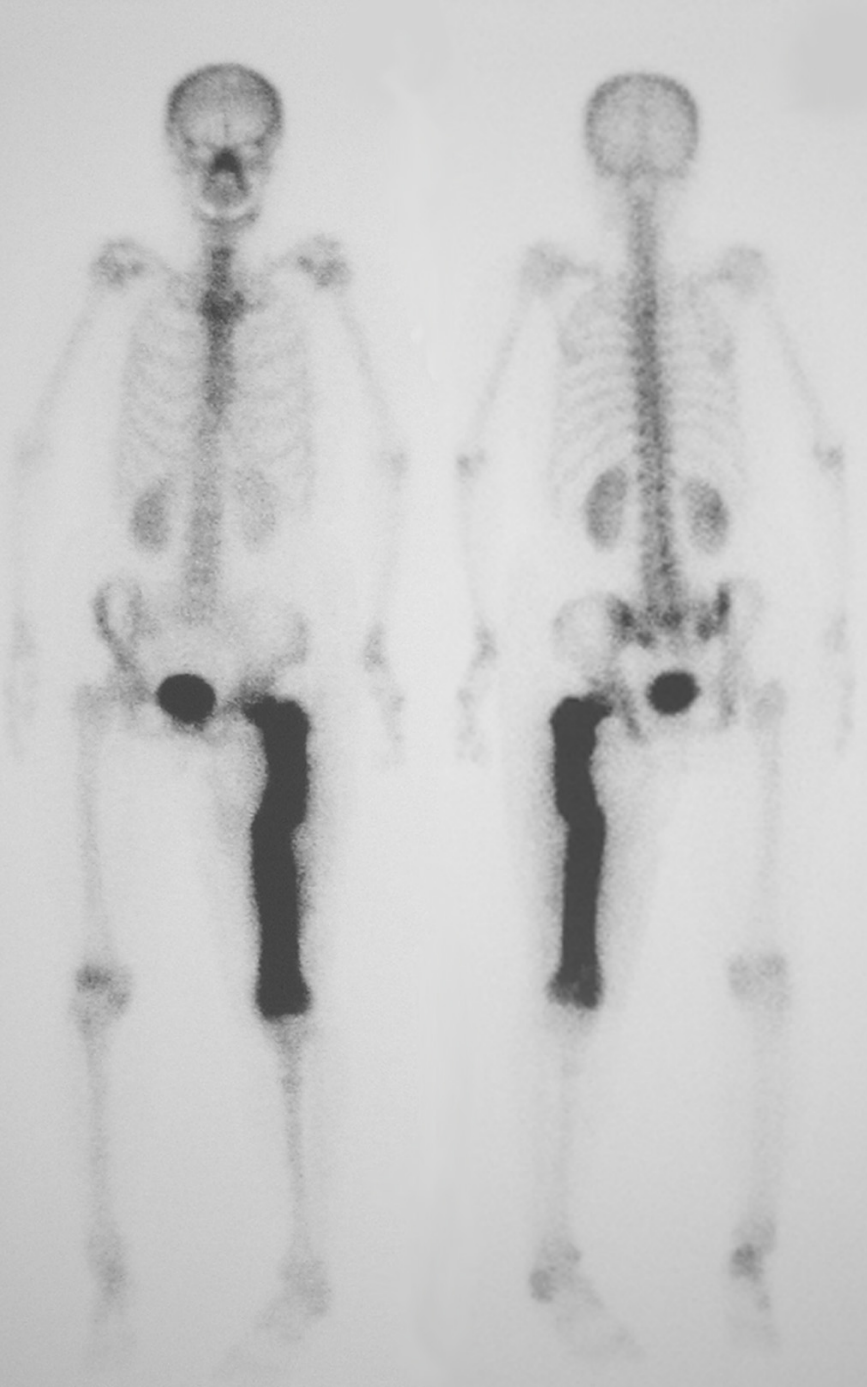
**Radionuclide bone scan showing markedly increased uptake affecting the left femur**.

**Figure 3:**
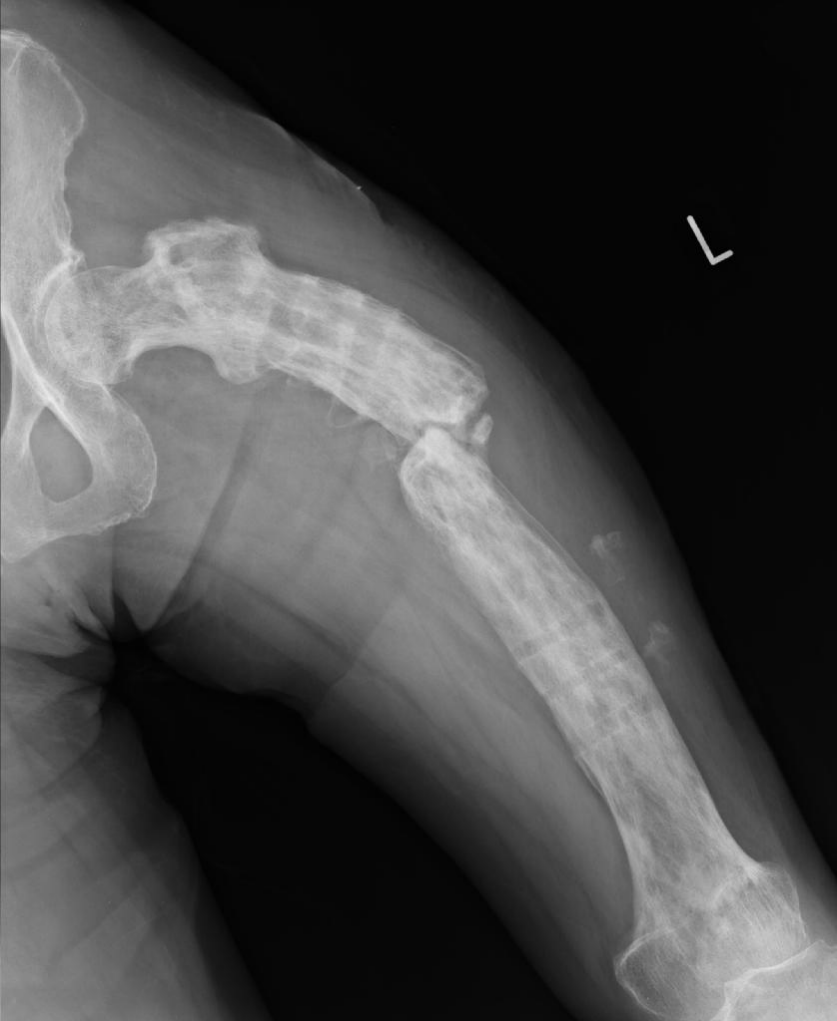
**Anteroposterior radiographic view 6 month after injury shows no sign of bone healing**.

**Figure 4:**
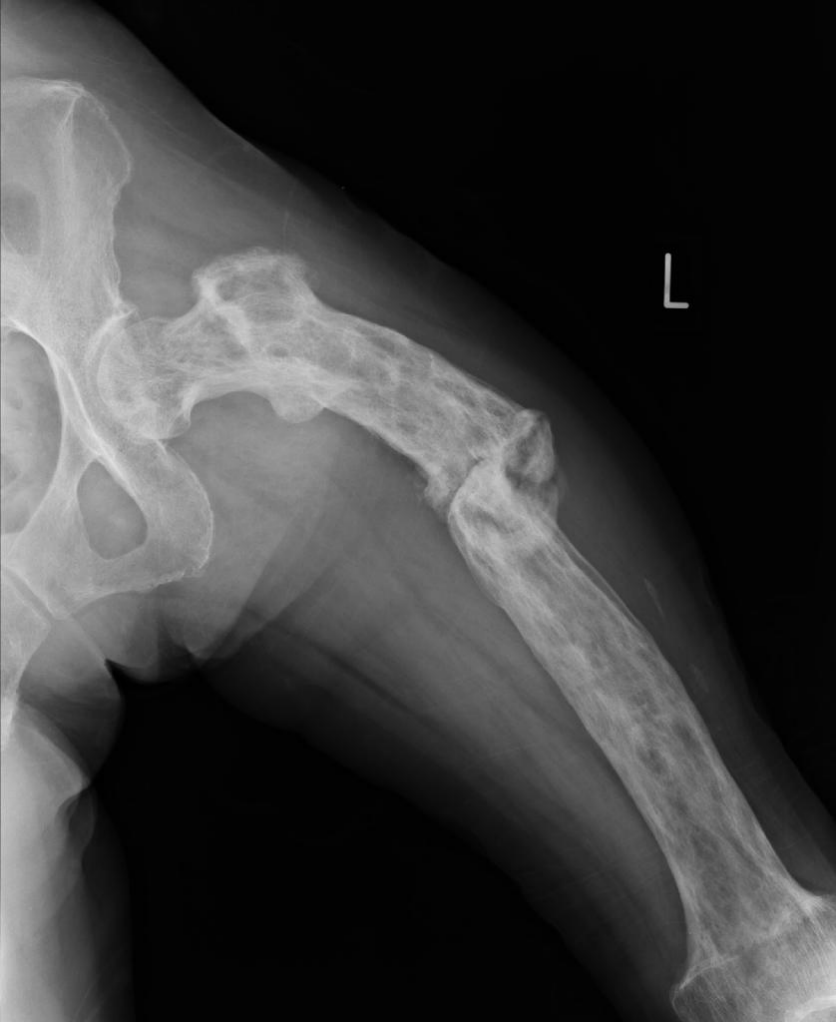
**Anteroposterior radiographic view showing fracture healing 6 months after application of the functional brace**.

**Figure 5:**
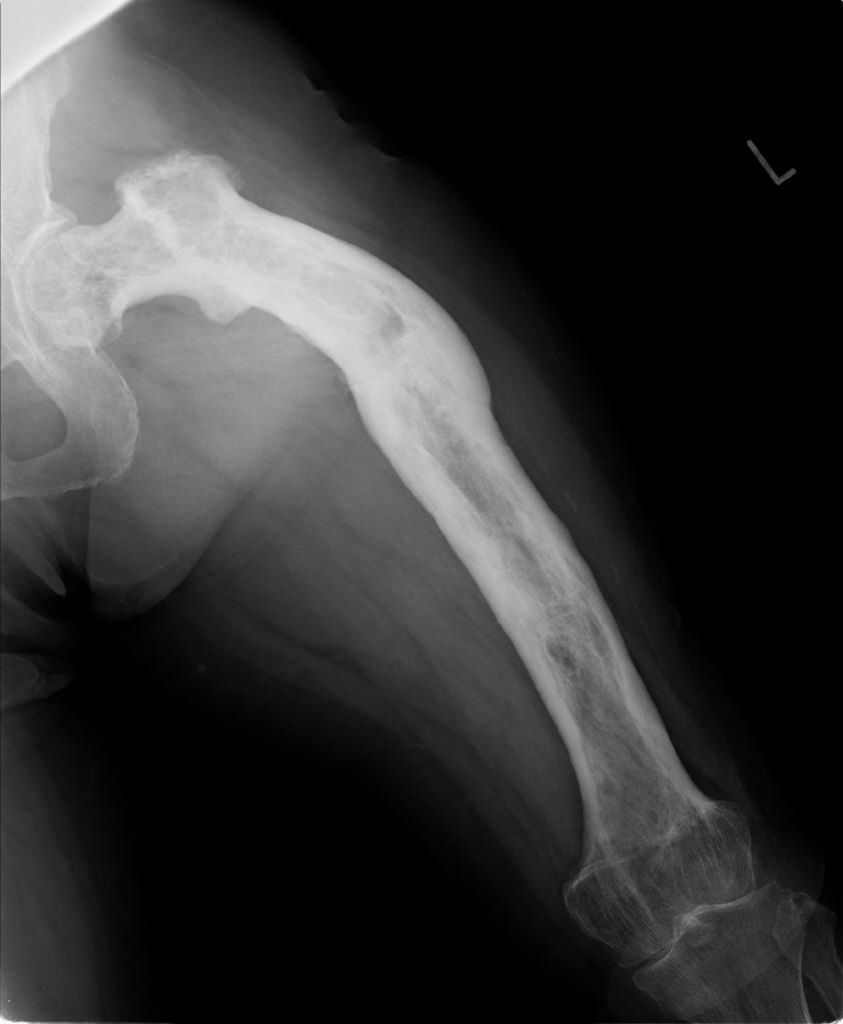
**Anteroposterior radiographic view 5 year after injury**.

The patient, a 63-year-old Japanese woman, presented at our hospital with severe thigh pain after suffering a fall. Plain radiography showed a displaced transverse fracture of the left femur (Figure 1). Osteosclerosis, osteolysis, enlargement, and bowing deformity were also noted in the femur. Laboratory tests revealed an elevated serum alkaline phosphatase level (455 IU/L; normal range: 115-359) with otherwise normal liver enzyme levels. Radionuclide bone scan showed dense uptake in the left femur (Figure 2). We diagnosed the patient as having pathological fracture secondary to monostotic Paget's disease. As she suffered from multiple concomitant illnesses, she was judged to be a poor risk for surgery. We therefore performed a closed reduction and stabilization with an external fixator. Later, however, we had to remove the external fixator because of infection at the pin site, and after 6 months of treatment there were no signs of bone healing (Figure 3). We diagnosed delayed union of the femur fracture, but surgical treatment for this situation could not be performed because of the patient's generally poor condition. We therefore applied a functional brace with the hope that the patient would be able to walk with crutches. X-ray revealed fracture healing after 6 months of treatment by functional bracing (Figure 4). At the latest follow-up 5 years after injury, there was complete healing of the fracture (Figure 5), the patient is able to walk unaided with a single T-cane.

## Discussion

Although Paget's disease of the bone is a relatively common disease in Australia, New Zealand, North America and most European countries, but it has a low incidence in Scandinavia, and is extremely rare in the Japanese population, with a prevalence of 0.15/100000; in patients aged 55 years of more, the proportion reaches 0.41/100000 [1-4,6]. The characteristic feature of Paget's disease is excessive bone resorption coupled with increased and disorganized bone formation. The affected bone is enlarged, disorganized in structure, and weakened. Pathological fractures are the most common complication of Paget's disease, and the treatment of such fractures is challenging. An increased rate of complications including delayed union, non-union, and malunion in pagetic bone fracture has been reported [8-10]. Open reduction and internal fixation of fractures has been recommended to prevent such complications[10]. However, plate and screw fixation requires extensive exposure, and in the present patient this was not possible because of her poor medical condition. Recently, there have been some reports of good fracture healing with the use of intramedullary nailing [11,12]. However, the latter is available only for mild bowing deformities. In the present patient, we decided to use an external fixator to fix this pathological fracture because of the above situation. However, after 6 months of treatment, the external fixator had to be removed due to pin site infection, even though fracture union had not been obtained. We then had no alternative but to apply a functional brace for delayed union of the femur fracture with the aim of allowing the patient to walk on crutches, although, to the best of our knowledge, no familial cases were found in the reported cases. Fortunately, in this case, fracture union was obtained 6 months after application of the functional brace. This treatment period is comparable to that reported by others using functional brace in the treatment of delayed union of the tibia [13-15]. We speculate that this treatment was advantageous because the external fixator and functional bracing did not violate the fracture site, allowing vascular regeneration and eliminating further damage to the peripheral and intramedullary blood supply which occurs during plate and screw fixation and intramedullary nailing. The success of this treatment suggests that functional bracing, a biological fracture treatment, may be a viable alternative for the treatment of fracture, delayed union, and non-union resulting from Paget's disease of the bone. This would be especially useful in the elderly and those considered at high risk from major corrective surgery. In recent years, the concept of biological osteosynthesis has gained a reputation in fracture treatment. Minimally invasive plate osteosynthesis (MIPO) techniques minimize the extent of soft tissue trauma to the injury zone, theoretically maintaining a better blood supply around the fracture area. Treatment of fractures secondary to Paget's disease using MIPO techniques might avoid the significant complications associated with more commonly used techniques of internal fixation.

This unusual case of delayed union of the femur fracture associated with Paget's disease of the bone for which functional bracing was ultimately successful illustrates the usefulness of biological fracture treatment in patients with this potentially refractory condition.

## Consent

Written informed consent was obtained from the patient for publication of this case report and any accompanying images. A copy of the written consent is available for review by the Editor-in-Chief of this journal

## Competing interests

The authors declare that they have no competing interests.

## Authors' contributions

IT has made substantial contributions to conception and design, or acquisition of data. AO, KM, MF, and KS have been involved in drafting the manuscript.

All authors read and approved the final manuscript.
